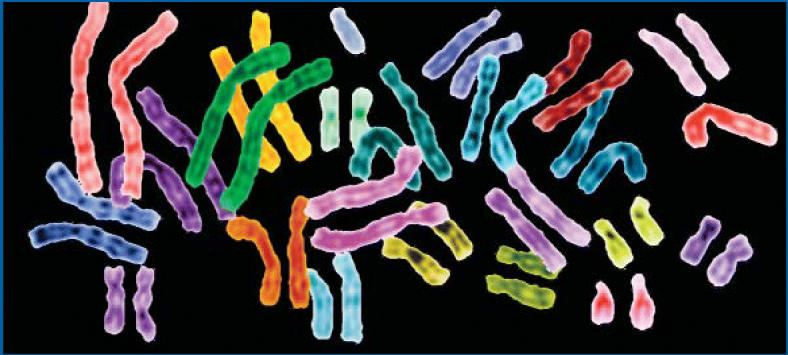# Headliners: Neurodevelopment: Genomewide Screen Reveals Candidate Genes for Neural Tube Defects

**Published:** 2006-06

**Authors:** Jerry Phelps

Rampersaud E, Bassuk AG, Enterline DS, George TM, Siegel DG, Melvin EC, et al. 2005. Whole genomewide linkage screen for neural tube defects reveals regions of interest on chromosomes 7 and 10. J Med Genet 42:940–946.

Neural tube defects are among the most serious and most common severely disabling forms of human birth defects. These defects—which arise from failure of the neural tube to close, an event that usually happens around day 28 after conception—are thought to be caused by a complex interaction between a person’s genetic makeup and environmental factors. Now NIEHS grantee Marcy C. Speer of Duke University Medical Center and colleagues from 14 research facilities across the United States report on a nationwide collaborative effort to gain new insights into the possible sites of a neural tube defect gene or genes.

There are three major types of neural tube defects, all with devastating consequences. Nearly all children with anencephaly (the absence of a major portion of the brain, skull, and scalp) die *in utero* or shortly after birth. Children with encephalocele (in which the brain protrudes through an opening in the skull) may survive but are almost always mentally retarded. And children with spina bifida (in which the spine fails to close properly) have varying degrees of muscle weakness and sensory disorders.

The most important environmental risk factor for neural tube defects is insufficient folate consumption by the mother around the time of conception. Folate supplementation reduces the risk of neural tube defect recurrence by 50–70%, but it does not entirely eliminate the risk. This suggests underlying genetic factors, a supposition bolstered by the increased rate of recurrence in siblings and the increased risk of defects in the offspring of a person with a neural tube defect. However, studies of folate-related and other developmental genes in humans have failed to definitively identify a gene causing neural tube defects.

In the current study, the researchers identified 44 families with more than one occurrence of a neural tube defect. They extracted DNA from whole blood samples of the affected individuals and related family members, for a total of 292 samples. Then they performed both parametric and nonparametric genomic linkage analyses. The results pointed to two candidate genes on human chromosome 7 and three on chromosome 10.

The researchers expect these results will help to prioritize future studies of neural tube defect candidate genes, and they plan to add additional families to their analyses. They also want to expand the phenotypic classifications to allow for a greater sample size and integrate other data such as those from mouse models of neural tube defects. The data in the present study bring the medical community closer to the day when individual-level prediction of the risk of a neural tube defect may be possible.

## Figures and Tables

**Figure:**